# Spectral Discrimination of Macronutrient Deficiencies in Greenhouse Grown Flue-Cured Tobacco

**DOI:** 10.3390/plants12020280

**Published:** 2023-01-07

**Authors:** Josh Henry, Patrick Veazie, Marschall Furman, Matthew Vann, Brian Whipker

**Affiliations:** 1 Department of Horticultural Science, North Carolina State University, Raleigh, NC 27606, USA; 2 Department of Statistics, North Carolina State University, Raleigh, NC 27606, USA; 3 Department of Crop and Soil Sciences, North Carolina State University, Raleigh, NC 27606, USA

**Keywords:** macronutrients, Hoagland solution, spectroscopy, principal component analysis, information entropy, classification modeling

## Abstract

Remote sensing of nutrient disorders has become more common in recent years. Most research has considered one or two nutrient disorders and few studies have sought to distinguish among multiple macronutrient deficiencies. This study was conducted to provide a baseline spectral characterization of macronutrient deficiencies in flue-cured tobacco (*Nicotiana tabacum* L.). Reflectance measurements were obtained from greenhouse-grown nutrient-deficient plants at several stages of development. Feature selection methods including information entropy and first and second derivatives were used to identify wavelengths useful for discriminating among these deficiencies. Detected variability was primarily within wavelengths in the visible spectrum, while near-infrared and shortwave-infrared radiation contributed little to the observed variability. Principal component analysis was used to reduce data dimensionality and the selected components were used to develop linear discriminant analysis models to classify the symptoms. Classification models for young, intermediate, and mature plants had overall accuracies of 92%, 82%, and 75%, respectively, when using 10 principal components. Nitrogen, sulfur, and magnesium deficiencies exhibited greater classification accuracies, while phosphorus and potassium deficiencies demonstrated poor or inconsistent results. This study demonstrates that spectral analysis of flue-cured tobacco is a promising methodology to improve current scouting methods.

## 1. Introduction

Remote sensing of nutrient disorders enables growers to rapidly scout their crops and manage fertilization practices with greater precision. Automation using spectral sensors mounted to trucks, tractor spray booms, or unmanned aerial vehicles (UAVs) is becoming more common in agricultural production settings [1,2]. Spectral sensors measure crop reflectance at various wavelengths in the electromagnetic spectrum, which can be correlated to the symptoms of specific nutrient disorders. Plants exposed to various biotic and abiotic stressors exhibit different visual symptoms, due in part to changes in how light interacts with plant tissues [1,3]. This characteristic enables spectral sensors to diagnose plant health problems before symptoms are visually apparent [3]. For instance, Osborne et al. [4] found several wavelengths in the visible and near-infrared (NIR) spectra that could be used to estimate nitrogen (N) content in field-grown corn measured under ambient light conditions. Zhang et al. [5] reported several wavelengths that were correlated with N, phosphorus (P), or potassium (K) deficiencies in field-grown rapeseed (*Brassica napus* L.) measured under artificial lighting within a laboratory setting. These studies demonstrate that spectral signatures can be correlated to specific nutrient disorders and can then be used to distinguish among disorders.

Certain wavelengths are already associated with plant stress in general and nutrient stress specifically. Many of these bands fall within the red edge, the portion of the spectrum between the visible red and NIR spectra. These wavelengths are located at approximately 700, 720, and 740 nm [6]. Other wavelengths of interest fall in the blue, (375, 466, and 490 nm), green (515, 520, 525, 550, and 575 nm) and red (675 and 682 nm) spectra [6]. These wavelengths have been used to classify nutrient stress or pigment changes [6]. Soil-Plant Analysis Development (SPAD) meters emit targeted electromagnetic radiation (EMR) near 650 and 940 nm and subsequently measure the amount of radiation transmitted through the leaf [7]. The values obtained from these sensors provide a measure of the green (high values) or yellow (low values) coloration exhibited in the foliage [7]. These values correlate to leaf chlorophyll concentrations which in turn correlate to leaf N concentrations. The primary goals of plant spectral analysis in terms of plant nutrition are the detection and discrimination of nutrient disorders or estimating foliar nutrient concentrations.

Rustioni et al. [8] reported successful discrimination among N, K, magnesium (Mg), and iron (Fe) deficiencies in greenhouse-grown grape (*Vitis vinifera* L.) leaves using hyperspectral reflectance measurements under artificial lighting. The effects of each deficiency on foliar pigment concentrations leads to the observed spectral differences [8]. Other studies by Adams et al. [9,10] demonstrate spectral separability of various micronutrient deficiencies. They found that copper (Cu)-deficient soybean [*Glycine max* (L.) Merr.] leaves from growth-chamber-grown plants were most successfully distinguishable, and manganese (Mn)-deficient leaves were also distinguishable in many cases; however, Fe and zinc (Zn) deficiencies were more difficult to classify [10]. Adams et al. [9] suggested that Cu, Fe, and Mn may affect spectral reflectance due to their specific roles in chlorophyll synthesis and electron transport.

Although there are at least 17 essential elements required to complete the cycle of plant growth and maturation, monitoring macronutrient status is considered most important for maintaining crop quality and yield. The essential macronutrients including N, P, K, calcium (Ca), Mg, and sulfur (S) are needed in relatively high concentrations [11]. Each of the essential nutrients perform crucial roles in plant development, and deficiencies of each nutrient cause unique symptoms that are visually diagnosable in many instances. Some of the primary effects of nutrient deficiencies include stunting and changes in leaf shape or orientation [12]. Nutrient deficiencies often cause changes in foliar coloration which may appear yellow (chlorotic), white (bleached), brown (necrotic), red, or black [12]. It is important to consider that the mobility of nutrients within plant tissues also dictate the location symptoms occur. For instance, deficiencies of mobile nutrients such as N, P, K, and Mg typically lead to symptom development on the mature lower foliage while relatively immobile nutrients such as Ca and S cause symptoms to develop on the upper foliage. Additionally, the pattern and distribution of symptoms on a single leaf are important to consider when diagnosing symptoms.

Henry et al. [13,14] induced nutrient disorders of flue-cured tobacco and provided detailed descriptions of the symptoms exhibited from each deficiency and toxicity. The results indicated that several nutrient disorders were readily induced, and symptoms had unique characteristics that made them visually distinguishable. Nitrogen deficiency appeared as a pale-yellow chlorosis that occurred uniformly on individual leaves but was more severe on the lower leaves [14]. Similarly, S deficiency led to symptoms of pale chlorosis; however, these symptoms occurred uniformly throughout the canopy [13]. Phosphorus deficiency resulted in symptoms of nonuniform chlorosis on the lower leaves with olive-green leaf spots and large necrotic lesions [13]. Potassium deficiency led to the development of marginal chlorosis on the lower to central leaves, while Mg deficiency caused symptoms of interveinal chlorosis in the same region of the plant [13].

Although numerous studies have been done to measure the spectral response of various nutrient deficiencies, fewer studies have compared the spectral responses of multiple nutrient deficiencies in one particular species. Besides nutrient status, numerous factors including plant species and maturity are known to impact spectral reflectance. Research on the effect of plant and leaf maturity on the spectral profile of healthy and nutrient deficient plants is limited. Studies investigating spectral reflectance in relation to foliar N concentrations in flue-cured tobacco have been conducted [15], but studies investigating multiple nutrient deficiencies in flue-cured tobacco have yet to be reported. Spectral remote sensing has significant potential to aid in modern crop production, especially for high-value crops such as flue-cured tobacco. Hyperspectral remote sensing can be used to diagnose plant nutrient status but is currently impractical to use on a commercial scale due to the excessive quantities of data and computational processing requirements. Using hyperspectral data to identify relevant wavelengths associated with particular nutrient deficiencies can lead to the development of relatively inexpensive sensors, offering greater opportunities for commercial utilization.

The purpose of this study was to determine if macronutrient deficiency symptoms could be distinguished among one another using spectral reflectance data. We hypothesized that discrimination of certain nutrient deficiencies would be more successful than others. In particular, we believed the symptoms of N and S deficiencies would be difficult to uncouple due to their visual similarities. This study was achieved by inducing N, P, K, Mg, and S deficiencies in a controlled environment and recording the spectral reflectance from each disorder at different stages of plant maturity.

## 2. Results

### 2.1. Deficiency Symptoms

**Figure 1:**
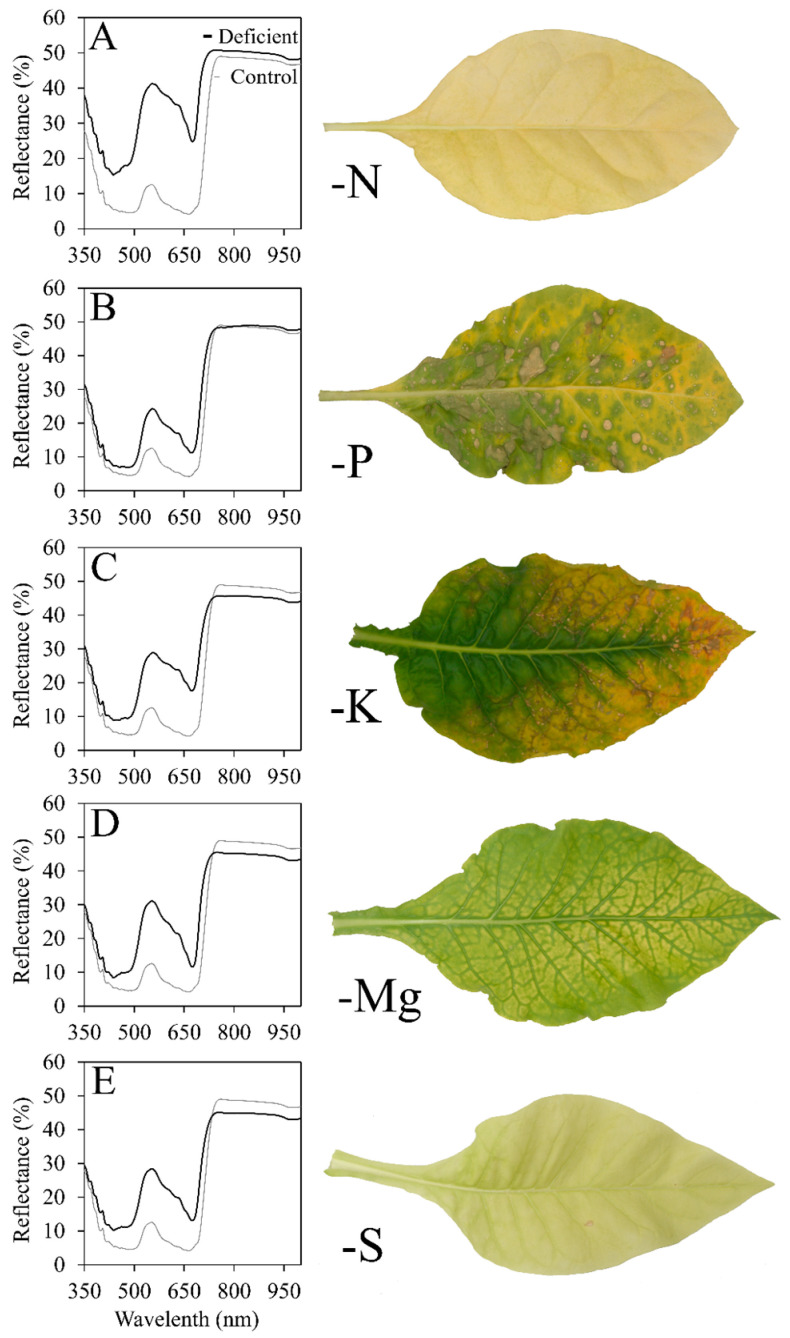
Average spectra and associated leaf appearance for flue-cured tobacco (*Nicotiana tabacum* L.) exhibiting: (**A**) nitrogen deficiency; (**B**) phosphorus deficiency; (**C**) potassium deficiency; (**D**) magnesium deficiency; (**E**) sulfur deficiency.

Plants grown in each nutrient deficient environment exhibited symptoms similar to those described by Henry et al. [13]. Nitrogen-, P-, and Mg-deficient plants always developed symptoms on the lower leaves first, regardless of growth stage. Young K-deficient plants developed symptoms on the lower leaves first, but mid- and late-stage plants exhibited symptoms primarily on the leaves within the middle third of the plant. Sulfur-deficient plants began developing symptoms on the upper foliage, spreading down the plant. Nitrogen-deficient (Figure 1A) and S-deficient (Figure 1E) plants both developed substantial chlorosis that eventually turned to complete foliar bleaching. Phosphorus-deficient plants developed nonuniform chlorosis with necrotic spotting (Figure 1B). Additionally, P-deficient leaves permanently lost their rigidity, appearing wilted with a noticeably pliable texture. Potassium-deficient plants developed the characteristic symptom of “firing” which consists of marginal chlorosis and necrosis (Figure 1C). Magnesium-deficient plants developed an interveinal chlorosis which turned to bleaching between the veins and downward-curling leaf margins (Figure 1D). Control plants consistently had higher foliar concentrations of the particular deficient nutrient than the nutrient-deficient plants at all maturity levels (Table 1).

### 2.2. Spectral Reflectance Characteristics

**Figure 2:**
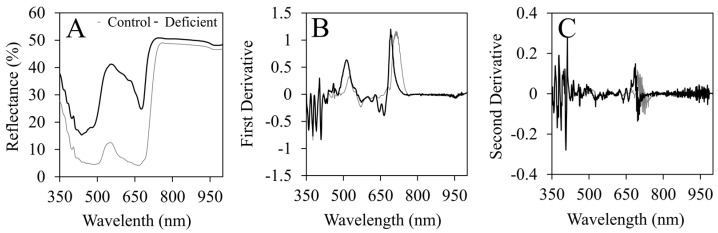
Graphs illustrating (**A**) the average spectral reflectance from 350 to 1000 nm for severely N-deficient flue-cured tobacco (*Nicotiana tabacum* L.) compared to the average control spectra, (**B**) the first derivatives of the N-deficient and control spectra, and (**C**) the second derivatives of the N-deficient and control spectra.

Average spectra of severely N-deficient leaves demonstrate greater reflectance than control leaves at every wavelength from 350 to 1000 nm (Figure 2A). Some of the major differences observed between N-deficient and control leaf spectra were observed in the green and yellow regions of the visible spectrum, with reflectance peaking near 550 nm at approximately 41.1% as opposed to 12.6% in control plants (Figure 2A). Control plants also exhibited similar reflectance of red (~650 nm) and blue (~450 nm) EMR, while N-deficient plants reflected higher quantities of red EMR proportionally (Figure 2A). Furthermore, raw spectral derivatives illustrated some of the primary areas of interest due to significant changes in slope. For instance, the first spectral derivatives for control and N-deficient plants both exhibited prominent peaks near 550 and 700 nm (Figure 2B). However, upon closer inspection, these two peaks illustrate the shift in the red edge from higher wavelengths in control plants to lower wavelengths in N-deficient plants (Figure 2B). These trends are also exhibited in the second derivatives, but less so and with significantly more noise, demonstrating the need to implement smoothing techniques (Figure 2C).

### 2.3. Band Selection

**Figure 3:**
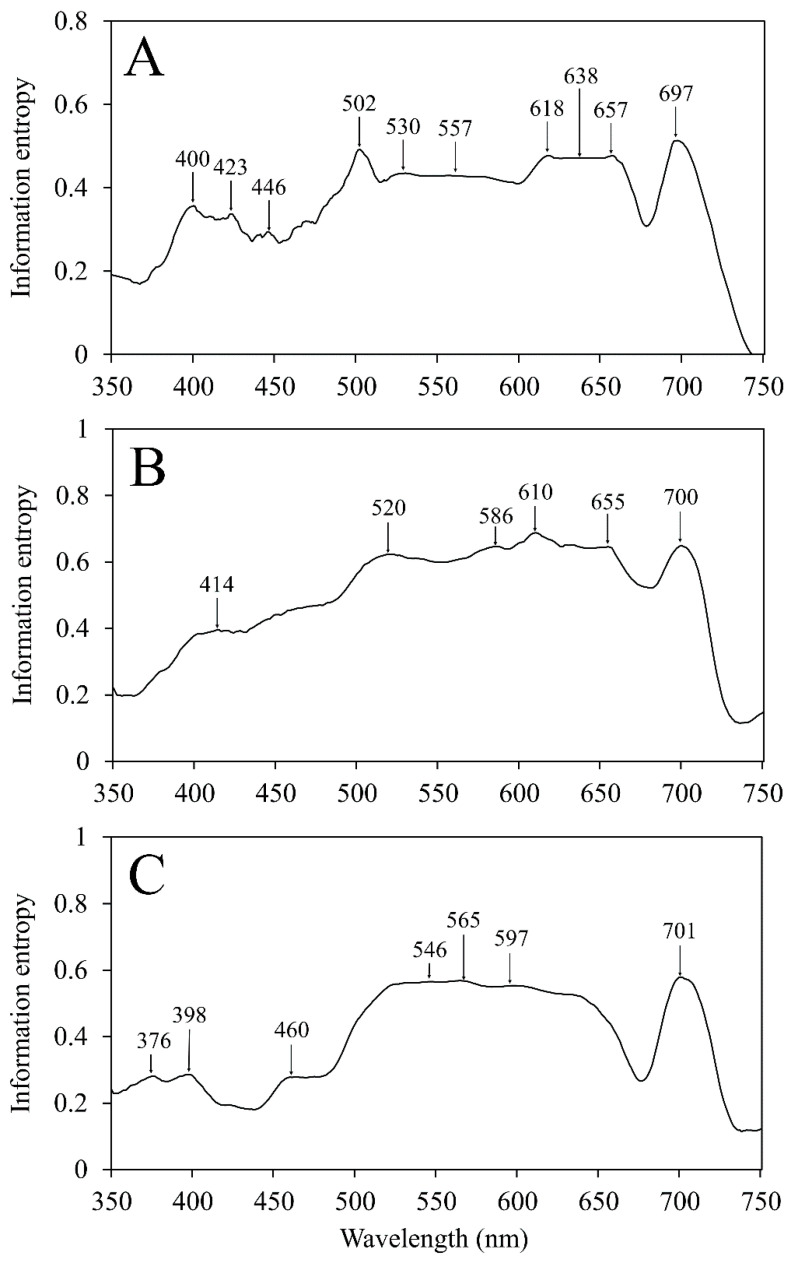
Savitzky–Golay-filtered information entropy observed among (**A**) young, (**B**) intermediate, and (**C**) mature flue-cured tobacco (*Nicotiana tabacum* L.) plants. Peak wavelengths demonstrate locations with the most informative bands necessary to distinguish among each nutrient deficiency.

Both information entropy and spectral derivative methods were able to successfully identify wavelengths that could explain the variability observed among symptoms (Table 2). In general, the majority of observed variability could be explained by wavelengths in the visible spectrum. Information entropy resulted in several peaks at 400, 423, 502, 530, 557, 618, 638, 657, and 697 nm for young plants (Figure 3A); 414, 520, 586, 610, 655, and 700 nm for intermediate maturity plants (Figure 3B); and 376, 398, 460, 546, 565, 597, and 701 nm for mature plants (Figure 3C). The highest peaks were typically between 500 and 650 nm, as well as a specific peak at 700 nm. Plants at each growth stage exhibited a similarly steep decreasing trend between 700 and 750 nm, coinciding with the red edge.

**Figure 4:**
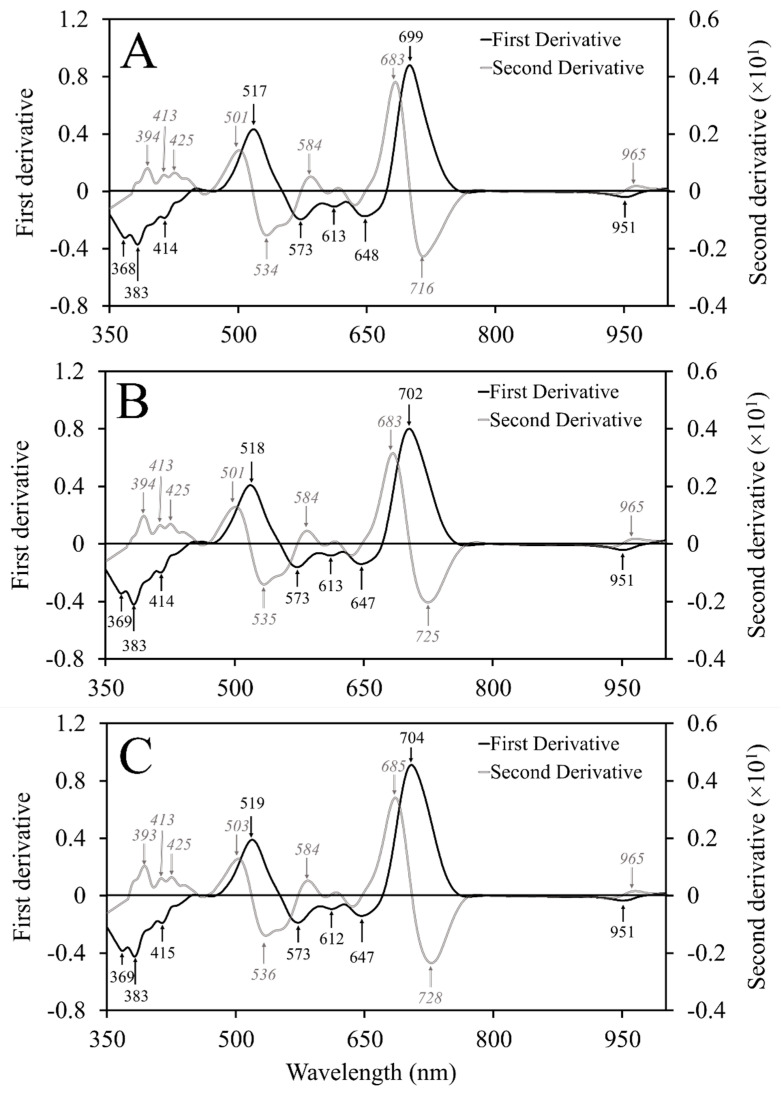
First and second derivatives of (**A**) young, (**B**) intermediate, and (**C**) mature flue-cured tobacco (*Nicotiana tabacum* L.) plants smoothed using the gap-segment algorithm. Peak wavelengths demonstrate locations with the most informative bands necessary to distinguish among each nutrient deficiency.

Several wavelengths were selected based on first and second derivative spectra (Table 2). Wavelengths selected from the first derivative spectra for young plants included 368, 383, 414, 517, 573, 613, 648, 699, and 951 nm (Figure 4A). Second derivative spectra resulted in the selection of 394, 413, 425, 501, 534, 584, 683, 716, and 965 nm (Figure 4A). Wavelengths selected from the first spectral derivative of intermediate maturity plants were 369, 383, 414, 518, 573, 613, 647, 702, and 951 nm (Figure 4B). Wavelengths selected from second derivatives included 394, 413, 425, 501, 535, 584, 683, 725, and 965 nm for intermediate maturity plants (Figure 4B). Finally, mature plants had selected wavelengths of 369, 383, 415, 519, 573, 612, 647, 704, and 951 nm for first derivative and 393, 413, 425, 503, 536, 584, 685, 728, and 965 nm for second derivative spectra (Figure 4C).

### 2.4. Symptom Classification

Principal component analysis (PCA) greatly reduced the data dimensionality and led to linear discriminant analysis (LDA) models with high classification accuracies. With just 10 principal components (PCs) used in each model, dimensionality was reduced by more than 95% as we were able to combine our original 2250 bands into 10 linear combinations of bands that captured most of the variability. This method was also found to successfully reduce dimensionality in other studies investigating plant spectral response [16,17]. Information entropy and PCA are both cited as useful methods for selecting bands containing the most information with regards to variability [16]. The LDA model for young plants resulted in 92% overall accuracy with individual accuracies of 92%, 100%, 88%, and 90% for control, N-deficient, P-deficient, and S-deficient plants, respectively (Table 3). The most common misclassification for young plants was between S deficiency and the control, with several S-deficient plants being classified as controls, and some controls classified as S-deficient.

Overall accuracy for intermediate maturity plants was lower than for young plants at 82%. Classification accuracies for control, N-deficient, P-deficient, K-deficient, Mg-deficient, and S-deficient plants were 94%, 92%, 58%, 70%, 79%, and 80%, respectively (Table 3). Nitrogen-deficient plants were successfully classified in most instances, but some N-deficient plants were misclassified as being Mg-deficient and vice versa. Additionally, a large proportion of P-deficient leaves were misclassified as control leaves. This may have been because P-deficient leaves had large green, chlorotic, and necrotic spots that likely resulted in more variable spectral measurements. Furthermore, a large portion of the upper foliage of P-deficient plants was visually asymptomatic, leading these leaves to be classified with control plants. 

Mature plants exhibited the lowest classification accuracy with an overall accuracy of 75%. Control plants had an 89% classification accuracy while N-deficient, P-deficient, K-deficient, Mg-deficient, and S-deficient plants had 100%, 52%, 42%, 92%, and 73%, respectively (Table 3). Potassium-deficient leaves were frequently misclassified as a control, Mg-deficient, or S-deficient leaf. Mature P-deficient leaves also exhibited low classification accuracy, with roughly one-third of all samples being classified as controls.

## 3. Discussion

Li et al. [18] studied hyperspectral reflectance for estimating N in upper, middle, and lower leaves of oilseed rape plants. They used partial least squares regression (PLSR) and reported optimal wavelengths of 437, 565, 667, 724, 993, 1084, and 1189 nm for upper leaves; 423, 570, 598, 659, 725, and 877 nm for middle leaves; and 420, 573, 597, 667, and 718 nm for lower leaves [18]. Li et al. [19] also used PLSR to model and predict foliar N and P concentrations in oilseed rape. They compared PLSR with other data transformations, such as first derivatives and continuum removal, and found that PLSR with first derivatives was most effective for nutrient estimation. The optimal wavelengths selected for N were 445, 556, 657, 764, 985, 1082, and 1994 nm while those selected for P included 755, 832, 891, 999, 1196, and 1267 nm [19]. Zhang et al. [5] investigated the spectral response of oilseed rape to N, P, and K deficiencies using PLSR. They reported optimal wavelengths of 440, 473, 513, 542, 659, 718, 744, 865, 928, 965, 986, and 1015 nm for N; 468, 522, 698, 721, 817, 967, 979, and 1025 nm for P; and 456, 554, 667, 720, and 1027 nm for K [5].

These past studies demonstrate similar results to what we obtained in the present study. In general, the majority of observed variation appears to be in the visible spectrum and falls specifically between 400 and 750 nm. However, other studies reported several significant wavelengths >800 nm [5,18,19], which was not the case in our study. Furthermore, we identified bands of interest in the UV region, whereas most other studies did not. This may be due in part to the specific spectroradiometer used in this study. It is also likely that the species and nutrient deficiencies used in this study were different from the other studies, and plants are known to demonstrate different spectral responses by species [6] and in response to different stimuli. Further work should investigate whether UV EMR is a good indicator of nutrient deficiencies in species other than tobacco.

Overall, PCA-based classification was highly accurate in distinguishing among the five macronutrient deficiencies induced in this study. Accuracy decreased with increasing maturity, likely due to the greater proportion of asymptomatic leaves on these plants. This proposed effect was especially impactful for the discrimination of P and K deficiencies. Nitrogen and S-deficient leaves were hypothesized to be similar enough to prevent accurate classification between these symptoms. However, in only three instances were S-deficient plants misclassified as N-deficient, and in no instance was a N-deficient plant misclassified as S-deficient. Phosphorus-deficient plants exhibited some of the lowest overall classification accuracies which may be attributed to the non-uniform appearance of the leaf surface. Similarly, mature K-deficient plants had poor classification accuracy, possibly because a large portion of the upper leaves appeared completely asymptomatic, and those leaves remained in the analysis. Therefore, it may be inferred that N-, Mg-, and S-deficient plants have a greater proportion of leaves that are visibly and spectrally different from the leaves of control plants. Future work may find that dividing leaves by visual symptoms could yield helpful insights.

## 4. Materials and Methods

### 4.1. Plant Material and Experimental Design

The experiment was replicated twice over time, and a total of 2563 individual sample readings were taken over the 2 trials. Each replication began by sowing pelletized K-326 tobacco seeds (GoldLeaf Seed Co., Hartsville, SC, USA) into 128-cell plug flats and placing them in a glass-glazed greenhouse at North Carolina State University in Raleigh, NC (35°47′41″ N lat, 78°41′57″ W long). The substrate was an 80:20 (*v*:*v*) mix of Canadian sphagnum peat moss (Conrad Fafard, Agawam, MA, USA) and horticultural coarse perlite (Perlite Vermiculite Packaging Industries, Inc., North Bloomfield, OH, USA), amended with mesh size #100 dolomitic limestone (Rockydale Agricultural, Roanoke, VA, USA) at 8.9 kg m^−3^ and wetting agent (AquaGro 2000 G; Aquatrols, Cherry Hill, NJ, USA) at 0.6 kg m^−3^. This custom substrate was used to limit nutrient contamination that would be present in a commercial substrate. Flats were irrigated by hand with nonfertilized water until seeds germinated and cotyledons fully expanded.

Following germination, seedlings were fertilized with a solution consisting of 7.5 mmol L^−1^ N, 0.5 mmol L^−1^ P, 3.0 mmol L^−1^ K, and 2.5 mmol L^−1^ Ca. Previous experiments demonstrated tobacco seedlings require a low concentration of primary macronutrients to develop sufficiently for studies investigating nutrient disorders [13,14]. The selected concentrations were a half rate of the primary macronutrient concentrations used in the nutrient-sufficient control solution. The control solution was a modified all-nitrate Hoagland solution consisting of 15.0 mmol L^−1^ N, 1.0 mmol L^−1^ P, 6.0 mmol L^−1^ K, 5.0 mmol L^−1^ Ca, 2.0 mmol L^−1^ Mg, and 2.0 mmol L^−1^ S, 72.0 µmol L^−1^ iron (Fe), 18.0 µmol L^−1^ Mn, 3.0 µmol L^−1^ Cu, 3.0 µmol L^−1^ Zn, 45.0 µmol L^−1^ boron (B), and 0.1 µmol L^−1^ molybdenum (Mo) [20] mixed with deionized (DI) water of 18 megohm purity. All nutrients were provided by the following technical grade salts (Fisher Scientific, Pittsburg, PA, USA): calcium nitrate tetrahydrate [Ca(NO_3_)_2_·4H_2_O], potassium nitrate (KNO_3_), potassium dihydrogen phosphate (KH_2_PO_4_), potassium sulfate (K_2_SO_4_), magnesium sulfate heptahydrate (MgSO_4_·7H_2_O), potassium chloride (KCl), calcium chloride dihydrate (CaCl_2_·2H_2_O), sodium nitrate (NaNO_3_), sodium phosphate dihydrate (NaH_2_PO_4_·2H_2_O), iron chelate (Fe-DTPA), manganese chloride tetrahydrate (MnCl_2_·4H_2_O), zinc chloride heptahydrate (ZnCl_2_·7H_2_O), copper chloride dihydrate CuCl_2_·2H_2_O, boric acid (H_3_BO_3_), and sodium molybdate dihydrate (Na_2_MoO_4_·2H_2_O). Solution pH was adjusted to ~6.0 using sodium hydroxide (NaOH). Macronutrient deficiencies were induced by replacing each cation (K^+^ and Mg^+^) or anion [(nitrate (NO_3_^−^), phosphate (H_2_PO_4_^−^), and sulfate (SO_4_^2−^)] containing salt with a sodium (Na) or chloride (Cl^−^) containing salt, respectively. Fertilizer treatments began upon transplanting into a sand culture system.

The seedlings were thoroughly drenched with DI water three consecutive times to leach any remaining nutrients prior to transplant. Seedlings were transplanted into 12.4 cm diameter pots (Dillen, Middlefield, OH, USA) filled with silica sand [Millersville #2 (0.8 to 1.2 mm diameter); Southern Products & Silica Co., Hoffman, NC, USA] that was soaked in sulfuric acid and triple-rinsed with DI water prior to use. The transplanted seedlings were placed into an automated recirculating sand culture system. The system was built on benches in a glass-glazed greenhouse in Raleigh, NC and utilized a completely randomized design. Each segment of the system (referred to as “lines” hereafter) consisted of 10.2 cm diameter polyvinyl chloride (PVC) piping (Charlotte Plastics, Charlotte, NC, USA) fit with 12.7 cm diameter PVC reducer couplings (Charlotte Plastics). Six couplings were used to hold pots and recapture irrigation solutions in each line. Fertilizer solutions were delivered via drip tubes fed from individual 20 L plastic buckets equipped with submersible pumps (model 1A; Little Giant Pump Co., Oklahoma City, OK, USA). Each line contained one treatment. Solutions were delivered for 1 min each cycle with as many cycles necessary per day to prevent apparent water stress (i.e., wilting) between 6:00 and 18:00 h. Nutrient solutions were replaced on a weekly basis.

### 4.2. Spectroscopic Measurements

A handheld spectrometer (PSM-2500; Spectral Evolution, Lawrence, MA, USA) was used to collect spectral reflectance data throughout the study. The sensor had a spectral range of 300 to 2500 nm and a spectral resolution of 3.5 nm at 700 nm, 22 nm at 1500 nm, and 22 nm at 2100 nm with the percent reflectance output in 1 nm increments. The sensor was equipped with a fiberoptic leaf clip capable of taking leaf-level measurements. This leaf clip had a self-contained light source with two light settings, of which the higher setting was used. Leaf clip measurements were taken inside the greenhouse under ambient light conditions. Measured plants were irrigated prior to measurement to ensure drought stress would not be a confounding factor.

Throughout the experiment, symptomatic and asymptomatic control plants were selected to obtain hyperspectral measurements of the individual leaves. At each measurement date, four individual plant replicates were selected. Reflectance data were collected from each leaf, except for the small bottom two to three leaves exhibiting natural senescence and the few immature upper leaves that were narrow with limited expansion. Measurements using the leaf clip were taken approximately 2 to 3 cm from the leaf margin and one-third of the leaf length away from the leaf tip. This location was selected because it typically represented the average degree of symptoms for each leaf. Areas of necrosis were avoided as necrotic tissues reflected much different patterns than non-necrotic tissues, regardless of symptomology.

### 4.3. Leaf Tissue Analysis

Leaf tissues were dried at 70 °C for 72 h and then ground in a sample mill (Thomas Wiley^®^ Mini-Mill; Thomas Scientific, Swedesboro, NJ, USA), and analyzed for nutrient concentrations (AgSource Laboratories, Lincoln, NE, USA). Total N was processed by Kjeldahl digestion and determined via flow injection analysis (FIA). Extractable K was processed by 2% acetic acid digestion and determined via inductively coupled plasma mass spectrometry (ICP-MS). Total P and all other plant minerals were processed by nitric acid/hydrogen peroxide digestion and determined via ICP-MS. Pairwise differences between control and deficient plant foliar nutrient concentrations were determined using PROC ANOVA in SAS (version 9.4; SAS Institute, Cary, NC, USA).

### 4.4. Data Preparation

**Figure 5:**
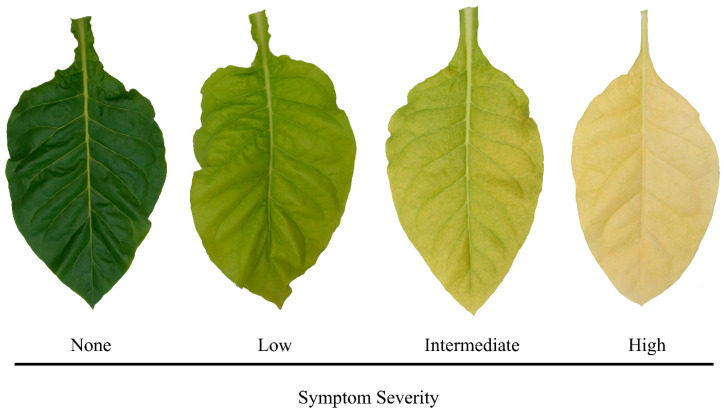
Demonstration of the flue-cured tobacco (*Nicotiana tabacum* L.) symptom severity rating system using nitrogen (N) deficiency as an example.

Reflectance data were manipulated and analyzed using R statistical software (R Core Team, Auckland, New Zealand). Measurements from each leaf of each treatment were assigned into several subgroups depending on various characteristics. Leaves received an objective symptom severity rating of four possible categories: (1) none, (2) low, (3) intermediate, or (4) high (Figure 5). We divided the symptom severity into four categories so that we could compare statistics for leaves that were visually symptomatic versus those that were not. Categorization did not affect the results of the study but allowed us to refine and better understand the symptoms that were observed. Examples of leaves from each category can be seen in Figure 5. Furthermore, measurement stages were separated by maturity: (1) young, (2) intermediate, or (3) mature. Young plants were those that had 6 or fewer leaves, intermediate plants had 7 to 12 leaves, and mature plants had more than 12 leaves. Fully mature tobacco plants developed between 18 and 20 expanded leaves prior to anthesis.

Water absorption bands were excluded from the analysis in the ranges of 1355 to 1450 nm and 1800 to 1950 nm. These wavelengths in the short-wave infrared (SWIR) region can be important in the detection of water related stress, but can also contribute noise, leading to the distortion of spectral measurements used for the detection of nutrient disorders [1].

### 4.5. Band Selection

Spectra were used to calculate information entropy as well as first and second spectral derivatives to identify which wavelengths contribute most to the variability observed among the nutrient-deficient leaves. Information entropy is a measurement for how much variability is contained within a band and it is commonly used for hyperspectral band selection [16,17,21]. Derivative measures are also useful for band selection by identifying areas with rapid changes in direction or pattern within spectra [16]. Information entropy was calculated using the FSelector package [22] while derivatives were calculated using the prospectr package [23] in R. Savitzky–Golay filtering was applied using a third-order polynomial with a smoothing window size of 10 bands to information entropy data to reduce noise and smooth the data. Derivatives were calculated and smoothed using the gap-segment algorithm with a filter length of 11 bands for both first and second order derivatives. Peaks and valleys were then calculated using the splus2R package [24] to determine the greatest individual absolute values or ranges of values within a span of 10 bands. Ranges of continuous values with uniform importance were aggregated and used to determine the central wavelength representing that range. The identified wavelengths represented those with the greatest impact on observed variability.

### 4.6. Symptom Classification

Reflectance spectra were analyzed by growth stage using PCA, with 10 PCs selected for each growth stage. Linear discriminant analysis was used to develop classification models to distinguish among the various symptoms. The classification models were conducted using a (custom-built) five-fold cross validation approach, where 80% of the data was randomly selected for model training and the remaining 20% used for testing. Following the first validation, a second validation was conducted with a new set of testing data previously used to train the model. This process was repeated to optimize the model until all data were used for both training and testing functions. The final classification results were put into a confusion matrix for each growth stage and analyzed using the PredPsych package [25] in R. The matrices were used to determine the overall accuracy and misclassification rates for each model. Overall accuracy was calculated by dividing the number of correctly classified observations by the total number of observations.

## 5. Conclusions

Spectral discrimination of nutrient deficiencies appears to be a promising form of precision scouting that should be investigated further in field scenarios. With continual advancements in sensors and greater availability of technology, spectral remote sensing has the potential to become an indispensable tool for agronomic crop producers. The wavelengths identified here and in other studies should be used to develop simpler and less-expensive sensors. Developing user-friendly software for automated spectral analysis should also be considered moving forward.

## Figures and Tables

**Table 1:** Pairwise comparisons of foliar nutrient concentrations found in the most recently matured leaves of young, intermediate, and mature flue-cured tobacco (*Nicotiana tabacum* L.) plants grown under macronutrient deficient conditions.

Treatment		-N	-P	-K	-Mg	-S
		Foliar nutrient concentration (%)				
Element		N	P	K	Mg	S
Young						
	Control	5.10a ^1^	0.41a	5.87	0.66	0.35a
	Deficient	0.86b	0.06b	—	—	0.10b
Intermediate						
	Control	3.17a	0.11a	4.46a	0.74a	0.32a
	Deficient	0.92b	0.05	0.34b	0.01b	0.09b
Mature						
	Control	2.47a	0.10a	3.37a	0.84a	0.33a
	Deficient	0.81b	0.04b	0.27b	0.01b	0.06b

^1^ Within column-ordered pairs grouped by plant maturity, means followed by the same letter are not significantly different according to Tukey’s HSD (0.05).

**Table 2:** Five most significant wavelengths (nm) selected for discrimination among macronutrient deficiencies in flue-cured tobacco (*Nicotiana tabacum* L.) at three growth stages. Band selection methods included information entropy, first spectral derivative, and second spectral derivative.

Maturity Stage		
Young	Intermediate	Mature
Information Entropy		
697	700	701
502	610	565
657	655	546
618	586	597
638	520	398
First Derivative		
699	702	704
517	518	519
383	383	383
368	369	369
648	414	573
Second Derivative		
683	683	685
716	725	728
534	535	536
501	501	503
394	394	393

**Table 3:** Linear discriminant analysis classification accuracies for nutrient deficient flue-cured tobacco (*Nicotiana tabacum* L.) plants based on principal component analysis.

Deficiency Treatment	Maturity Stage		
	Young	Intermediate	Mature
Control	92%	94%	89%
Nitrogen (N)	100%	92%	100%
Phosphorus (P)	88%	58%	52%
Potassium (K)	—	70%	42%
Magnesium (Mg)	—	79%	92%
Sulfur (S)	90%	80%	73%
Overall accuracy	92%	82%	75%

## Data Availability

The data presented in this study are available on request from the corresponding author.
